# Electronic health records, the device paradigm, and the need for engagement

**DOI:** 10.1186/s13010-025-00189-9

**Published:** 2025-09-30

**Authors:** Will Lyon

**Affiliations:** Department of Medicine, Division of Geriatric and Palliative Medicine, Medical College of Wisconsin, Milwaukee, USA

**Keywords:** Philosophy, Technology, Electronic health record (EHR), Clinical skills, Doctor-patient relationship

## Abstract

In *Technology and the Character of Contemporary Life*, Albert Borgman puts forth the “device paradigm” as characteristic of the way we interact with the world in our technological society. He argues that devices, while liberating and disburdening us from some effort, also result in a lack of physical and social engagement. In this essay I apply Borgman’s device paradigm to the electronic health record as an example of the device paradigm in healthcare, and argue that engagement and caring, two essential components of the doctor-patient relationship, are harmed by the EHR.

## Introduction

In a 2018 *New York Times* article, Abraham Verghese recalled his emergency room stay for an asthma attack that he suffered while traveling [1]. Verghese, an internal medicine physician, describes how he felt like a bystander to interactions between his nurse and the computer screen during the stay: “The nurse came in regularly, but not to visit me so much as the screen against the wall. Her back was to me as she asked, ‘on a scale of 1 to 10, with 10 being great difficulty breathing…?’” He uses this anecdote to convey his concerns about the lack of presence and human interaction that have resulted from widespread adoption of electronic health records (EHR).

In this essay I aim to demonstrate that by becoming enmeshed in a technological and industrial framework, doctors have lost important skills and virtues that are necessary for the work of healing. To make this case, I first describe Albert Borgmann’s “device paradigm” from *Technology and the Character of Contemporary Life* [2]. I then consider ways in which the device paradigm is apparent in the modern healthcare industry, and what human goods these devices may be replacing. Specifically, I focus on the EHR as an instance of the device paradigm in medicine and consider the consequences of the EHR for the practice of medicine. I argue that the EHR results in the loss of two important components of the relationship between practitioners and patients: engagement and caring.

### The device paradigm

In Borgman’s *Technology and the Character of Contemporary Life*, he seeks to “provide an explicit account of the pattern or paradigm of technology” [2]. For Borgmann, the pattern of technology is most clearly seen in what he calls the device paradigm. A device makes certain goods *available*. When using the term “available,” Borgmann means that the good in question has been “rendered instantaneous, ubiquitous, safe, and easy” [2]. Further, a device makes goods available through technological means in a way that is neither context-specific (i.e. it is separate from the context in which that commodity was procured in the pretechnological setting) nor burdensome.

As an example of the device paradigm, Borgmann provides the case of central heating as opposed to the traditional fireplace or hearth. The good or commodity produced by either of these is warmth. But “a device such as a central heating plant produces mere warmth and disburdens us of all other elements. These are taken over by the machinery of the device” [2]. A central heating unit makes heat *available* in the sense defined above. Prior to this specific device, heat was not available because it was not instantaneous (wood had to be split, a fire had to be built, etc.), ubiquitous (a fireplace only heated certain parts of the house), safe (one dealt more directly with fire and the fire was more apt to spread), or easy (“work, some skills, and attention were constantly required to build and sustain a fire”) [2].

To clarify these concepts, Borgmann distinguishes between devices and things. He states, “a thing, in the sense in which I want to use the word here, is inseparable from its context, namely, the world, and from our commerce with the thing and its world, namely engagement. The experience of a thing is always and also a bodily and social engagement with the thing’s world” [2]. Because of the engagement that is required, “a thing necessarily provides more than one commodity” [2]. In the case of the fireplace, it produces more than mere warmth, as Borgmann describes in moving, if nostalgic, detail:

“It was a focus, a hearth, a place that gathered the work and leisure of a family and gave the house a center. Its coldness marked the morning and the spreading of its warmth the beginning of the day. It assigned to the different family members tasks that defined their place in the household. The mother built the fire, the children kept the firebox filled, and the father cut the firewood. It provided for the entire family a regular and bodily engagement with the rhythm of the seasons that was woven together of the threat of cold and the solace of warmth, the smell of wood smoke, the exertion of sawing and of carrying, the teaching of skills, and the fidelity to daily tasks.” (p. 41–42).

So, a thing can be distinguished from a device by (a) the availability of the commodity in question (a device makes a commodity available, while a thing requires attention, skill, and work to produce a commodity); (b) the engagement involved in procuring the good in question (devices do not involve engagement, while things do); and (c) the number of commodities produced (a device tends to produce only one commodity, while a thing produces multiple commodities due to the engagement involved).

The concept of engagement is important in understanding Borgmann’s account of the way in which devices have changed the pattern or character of contemporary life. Defined as “our commerce with the thing and its world,” engagement takes place on multiple planes. Borgmann defines physical engagement as “not simply physical contact but the experience of the world through the manifold sensibility of the body” [2]. Beyond physical engagement, engaging with things involves commerce with the entire context or world of that thing, including its social, material, cultural, and other contexts. The importance of engagement for Borgmann’s account is that the adoption of devices leads to decreased engagement, and therefore loss of the other goods that resulted from that engagement.

In the above I have presented Borgmann’s device paradigm, which he does not specifically define but instead illustrates through examples and introduction of more concepts. Before moving on, I want to call out three points made by Borgmann in his discussion of the device paradigm and the effects of devices which will be relevant to the rest of this essay. The first is the concept of *engagement*, which was clarified in the preceding paragraph. The second is the observation that things produce manifold commodities, while devices produce a single commodity. Third, the disburdening tendencies of devices mean that they make “no demands on our skill, strength, or attention” [2]. In what follows I will attempt to apply Borgmann’s device paradigm to the role of EHRs in medicine.

### The electronic health record and the device paradigm in medicine

The use of EHRs is both new and widespread in the healthcare industry – 78% of office based physicians and 96% of acute care hospitals in the United States employed an EHR in 2021. In 2008, these percentages were 17% and 9%, respectively, showing how rapid the adoption of EHRs has been [3]. The name “electronic health record” is something of a misnomer for the software programs used by doctors, nurses, medical assistants, and other clinical staff to conduct their daily tasks. Most EHRs are much more than a record keeping software. They typically integrate manifold functions including scheduling, messaging and notifications, electronic order entry, and clinical decision support into the interface used by the clinician.

The EHR, and each of its components or functions described above, is a device in Borgmann’s sense of the term, namely that it renders certain commodities available (instantaneous, ubiquitous, safe, and easy). Given the complexity and multi-functional nature of modern EHRs, and the scope of this essay, I will focus primarily on the record-keeping function of the EHR, since this is at least one feature that is consistent between different software programs and interfaces. In this case, the commodity that is made available is information about the patient, including their medical and surgical history, notes from other clinicians, lab and imaging results, currently prescribed medications, among other important information. This information can be accessed instantaneously, nearly ubiquitously (provided one has a computer and an internet connection), safely (more on that later), and easily. Apart from accessing information, the EHR also provides an interface for entering and documenting information (although it is unclear if this has made documentation easier, since clinicians now spend more time than ever on documentation).

As the scale and ubiquity of the EHR has grown, and EHR platforms have become more synchronized, patient data across decades-long periods and between different health systems and states becomes easily accessible to the clinician. This has disburdening and liberating effects when it comes to obtaining accurate (presumably) and consistent data about patients. Prior to EHRs, it was laborious to find records, obtain them from other locations, manually review them for the desired data, and organize and store them. EHRs also liberate the clinician from the time-consuming process of confirming all medical history with a patient when the relationship is new (although, as we will see, an over-reliance on the EHR can lead to perpetuation of inaccurate medical information). In this way, the EHR has improved the efficiency and resource optimization of clinicians when it comes to record keeping and health information.

For Borgmann, when a commodity is made available by a device, it takes away various forms of *engagement* with the world of things (defined above). Remember that for Borgmann engagement is not only physical but involves commerce with the broader social, natural, and cultural contexts of the thing. When patient history is made “available” by data presented by the EMR, and the clinician takes this information at face value, he forfeits an engagement with the world of the person suffering illness and seeking healing. Consider the following case, presented by Steven McGee [4]:

A doctor on call is paged by the emergency room for a new patient to be admitted to their medical service. The doctor briefly reviews the EHR before going to meet the patient in the ER. He sees that the patient has been admitted three times in the last six weeks due to what has been called end-stage cirrhosis. When the physician examines the patient,

“There are the expected signs of liver disease—spidery blood vessels on his face and neck, distended abdomen, and swollen legs—but one finding is completely unexpected. Just beneath the patient’s right ear, quick inward pulsations of the soft tissues are conspicuous, beating twice per heartbeat. In an instant—what seasoned clinicians call the “Augenblick” or “blink-of-an-eye” diagnosis—the physician knows the patient’s problem is not cirrhosis. The venous pulsations in the patient’s neck indicate the diagnosis is constrictive pericarditis, a disorder in which the heart has become encased in scar tissue, causing the patient to retain fluid. The patient needs surgical removal of his diseased pericardium” [4].

McGee goes on to consider why this diagnosis was missed during the first three hospitalizations, and concludes that decline in physical exam competency is a major contributor. Parallel to this is an over-reliance on the information presented by the EHR, rather than information presented to our senses by the patient themselves. In the setting of this over-reliance on electronic health information, practical wisdom and bedside medicine become secondary to “chart review” and clinical decisions based on EHR data.

### EHRs and the flipped patient

I have just described the way in which the EHR fits with Borgmann’s device paradigm, before introducing concerns about the engagement that is forfeited by an over-reliance on this device. In *Clinical Education and the Electronic Health Record* [5], Abraham Verghese describes the phenomenon of the “flipped patient” (or, as he calls it elsewhere, the “iPatient”). The flipped patient phenomenon occurs when the initial encounter a physician has with a patient is with the digital representation of that patient present in the EHR, rather than with the flesh-and-blood patient. While reviewing the EHR before seeing a patient provides important opportunities to become familiar with that patient’s recent clinical data, general history and opportunities for rapport building, Verghese explains, “In meeting the iPatient first, the medical student no longer needs to ask the time-honored question, ‘What brings you to the hospital today?’ The presenting problem and a preliminary diagnosis have already been entered among a computerized list of medical problems” [5]. Verghese raises concerns that this will lead to a decline in clinical skills including patient interview, physical exam, and moral traits including patience, attentiveness, and empathy.

While presenting multiple possible reasons for the flipped patient phenomenon, Verghese’s main concern is that students and resident physicians trained in this environment will become more comfortable in front of a computer screen and less comfortable with patients. Further, false information can become perpetuated in the EHR as “chart lore.” This becomes problematic, especially because certain diagnoses can only be made based on history and physical examination [6]. Ultimately, Verghese concludes, “the nature of medicine is the interaction of a vulnerable human being in distress with a caring empathetic team represented by other humans. It is vital to set EHR guidelines during training to foster skill in getting to know and care for patients” [5].

This “interaction” that Verghese bemoans the loss of qualifies as a type of engagement on Borgmann’s account. Recall that Borgmann describes physical engagement as “not simply physical contact but the experience of the world through the manifold sensibility of the body,” and that engagement also involves commerce with the social, material, and cultural contexts of that thing [2]. This deep interaction involves a combination of attention, care, and skill which is not asked of us when the device paradigm is in play. Thinking back to Borgmann’s fireplace example, it would be reasonable to draw a parallel between the hearth and the physician’s history-taking and physical exam as examples of engagement, and another parallel between central heating and the EHR as examples of devices.

While the liberating and disburdening effects of EHRs are clear, the cost in terms of loss of engagement is a real one. This is because certain virtues and skills are essential to the work of healing. These include caring, attention, and competency in physical diagnosis. As I have already considered the last of these skills in the current section, I will now shift our focus to the virtue of caring (I do not treat attention in detail here, but it is clearly a prerequisite for caring, and I have discussed attention elsewhere in the context of EHRs) [7].

### Care, means, and engagement

In the above anecdote from Dr. Verghese’s ER visit, he summarized the experience as follows “I received care but did not feel cared for” [1]. The virtue of care, or caring, is “a fundamental and directional virtue of relationships, practices, and actions in health care” [8]. The language and virtues associated with caring (attentiveness, empathy, fidelity) are strongly intertwined with the practice of medicine, and an entire moral approach – that of *care ethics* – was developed in response to an over-reliance on language of rights, justice, and other abstract moral concepts. As Beauchamp and Childress summarize, caring refers to a “care for, emotional commitment to, and deep willingness to act on behalf of persons with whom one has a significant relationship… The ethics of care emphasizes not only *what* physicians and nurses do… but also *how* they perform these actions” [8]. For the ethics of care, the practitioner’s actions are important but so are their dispositions and intentions and the effects of their actions on the relationship. Put another way, the means are important, and not only the ends.

For Borgmann, a characteristic feature of devices is that they obscure the *means* by which a commodity is produced. The means become obscured from view and are no longer context-specific. When the flipped patient phenomenon occurs, it becomes easy to conflate the digital abstraction of the patient presented by the EHR with the real person. But to do so is to take the clinical information out of context. When the practitioner becomes too trusting of the information in the EHR, and relies more on chart data than on engagement with the patient, *commodities* such as diagnoses and treatment plans are made *available* without face-to-face interaction with the patient. This is how a patient with a strangulated inguinal hernia can instead be misdiagnosed with “food poisoning” and a patient with shingles on their chest can be taken to the cardiac catheterization lab for treatment of a suspected heart attack [9]. In these unfortunate cases, the *means* by which a diagnosis or therapeutic plan was formulated was separate from the context of the patient’s story and body. To an extent, the EHR facilitates this type of error by creating the illusion of clinical decision making by neatly organizing and presenting clinical data. Even in instances not related to frank diagnostic or therapeutic errors, a lack of rapport and engagement between doctor and patient may result in decreased cooperation with prescribed treatment plans.

The consequences of the EHR introduced thus far include a loss of engagement and a weakening of caring and its related virtues. Based on Borgmann’s account, these consequences are to be expected due to the disburdening effects and the separation of means and ends brought about by devices. While these effects may be desirable or irrelevant in the case of some technological devices, e.g. a dishwasher, they are significant in the practice of medicine because the engagement and care are diverted from the suffering person, which ought to be the focal point of both clinical medicine and medical ethics.

One might object that the lack of engagement and caring are individual moral and clinical failures, and not a consequence of EHR software, since EHRs are simply for record keeping and electronic order entry. On this objection, my description of diagnoses and treatments as “commodities” made available by EHRs misrepresents the function of EHRs and downplays the decision of practitioners to rely more on chart data than thoroughly evaluate the patient. But the systemic and total implementation of EHRs for almost all patient-care-related tasks has led to a system in which individual practitioners are left little choice but to spend most of their time in front of a EHR rather than with patients [10]. Considering that careful bedside assessment and building a doctor-patient relationship take time, patience, and attention, performing these tasks well comes at the cost of more time spent completing required EHR-related tasks and in this way, time with the patient can become *de facto* disincentivized.

In this essay, I have presented the “device paradigm” based on Albert Borgmann’s account and made the case for the EHR as an instantiation of the device paradigm in healthcare. I have considered the deleterious effects of this, employing Borgmann’s concept of engagement as well as considering the ethics of caring. Currently, some writers argue that artificial intelligence and machine learning can take over documentation and other EHR tasks that consume most of physicians’ time. Further work should consider whether this might allow for more engagement with and attention paid to patients, or whether this will exacerbate the concerns elaborated above by perpetuating inaccurate or incomplete information within patient’s records and by providing more diagnostic and treatment recommendations that allow practitioners to forego engagement with patients.

## Data Availability

No datasets were generated or analysed during the current study.

## Abbreviations

HER: Electronic health record
